# A Retrospective Community Perspective: Bridging the Health Disparity Gap with Obesity as the Leading Comorbidity in Disparities in Health Outcomes in the COVID-19 Pandemic

**DOI:** 10.21203/rs.3.rs-2043805/v1

**Published:** 2023-01-27

**Authors:** Odiase Peace, Henry Terry, Amita Banga, Kartik Rachakonda, Amar P Garg, Girish Rachakonda

**Affiliations:** Meharry Medical College; Meharry Medical College; Shobhit University; University of South Florida College of Arts & Sciences; Shobhit University; Meharry Medical College

**Keywords:** associated risk of coronavirus with comorbidities, SARS-CoV2, COVID-19, diabetes, cardiovascular disease, health disparities

## Abstract

COVID-19 is a viral infection that resulted in a global pandemic. In the United States, COVID-19 caused incommensurate deaths, especially among members of minority groups. Previous literature shows comorbidities such as hypertension (HTN), diabetes mellitus (DM) and obesity (OBS) have been implicated in the severity of COVID-19 cases regardless of racial or ethnic group classification. However, minority populations, particularly people of African descent experienced higher mortality as they carry a disproportionately heavier burden in comorbidities cases. In this study we first confirm current literature on COVID-19 incidence and its correlation with the prevalence of comorbidities in various racial and ethnic populations, using anonymous and aggregated data from the Nashville General Hospital at Meharry, an Institute for the Study of Minority Health. We also evaluated the prevalence of comorbidities in minority patients and computed the correlation between the COVID-19 incidence and a permuted prevalence of comorbidities. A total of 959 patients were reviewed and our study indicates COVID-19 patients classified as Non-Hispanic Blacks (NHB) were approximately 3 times more likely to have an HTN or DM or both HTN and DM diagnosis. The chances double to be approximately six times higher when an OBS diagnosis is included singularly or in conjunction with either HTN or DM or both HTN and DM.

## 1. introduction

Health disparities refer to avertible differences in the incidence, health outcomes, and burden of diseases among populations [1]. There are large racial and ethnic differences in health outcomes in the United States and investigators are seeking to link prevalent differences in a specific health condition or disease within an identifiable population group to differential exposure to risk factors and resources including diet, lifestyle, and psychosocial, physical, and chemical components [2]. In the United States, disproportionate mortality has been observed among ethnic minorities in 2020 during COVID-19 pandemic [3]. Interestingly, disparities in the comorbidities frequently associated with COVID-19 already exist within the population. For example, Blacks or African Americans are 30% more likely to die from heart disease, twice as likely to have a stroke, and show a higher rate of Myocardial Infarction, heart failure, functional impairment from Acute Coronary Syndrome (ACS), death rate from ACS and high blood pressure [4]. They are also 10% less likely than their white counterparts to have their blood pressure under control, twice as likely to be diagnosed with Diabetes and have higher rates of peripheral vascular disease and higher rates of Obesity [4].

The combined knowledge from these studies highlights the potential that ethnic minorities such as Blacks or African Americans with at least one comorbidity, such as hypertension (HTN), Obesity (OBS), coronary heart disease, lung problems, and diabetes mellitus (DM) present are at an increased risk of severe infection with coronavirus and a consequent higher mortality rate. The association between comorbidities and the increased fatality rate for COVID-19 among reported multi-racial population groups provides an alternative approach for analyzing the current data. In this study, we investigated the correlation between co-morbidities in racial groups and COVID-19 diagnosis using the anonymized data available from Nashville General Hospital at Meharry. We looked at the preexisting characteristics of patients admitted into the Meharry Medical Facilities for the specific comorbidities known to contribute to the increased severity of COVID-19. Then, using the existing patient population at Meharry we observed and analyzed the rate of COVID-19 infection and the prevalence of comorbidities across racial groups to find an association. The information we uncover can be translated to improve the assessment of patient risk and optimize care in clinical settings, especially in the face of new variants.

## 2. Materials And Methods

This retrospective study included anonymous and aggregated data from the Nashville General Hospital (NGH) at Meharry, an Institute for the Study of Minority Health from May 2020 to June 2021. The Clearsense Discovery Database at Meharry is a data management platform that hosts de-identified electronic health records data (EHR) from NGH, a health center that provides clinical services to safety-net populations such as individuals with low-income who are uninsured or under-insured and racial/ethnic minorities [5]. Relevant data elements retrieved from the dataset encompassed diagnoses and racial demographic statistics.

We retrieved patient data and clustered them into different cohorts based on the criteria met. Cohort 1 patients were patients with a newly presenting COVID-19 diagnosis using the admission code ICD-10-CM for confirmed COVID-19 diagnosis from May 2020 to June 2021. Cohort 2 was composed of patients with the following comorbidities: HTN, DM, and OBS, which are independent factors known to be associated with increased severity of COVID-19 infection. In our exploration for cohort 2, the ICD-9-CM code was used to search for: Diabetes, and diseases of the circulatory system including ischemic heart disease, heart disease, HTN, and OBS without a specific time frame. Cohort 3 was composed of all patients admitted into the system without a specific time frame and without preference for an admission type. Admission types referred to patient visit categories such as outpatient, emergency, long-term care, and inpatient visits. Cohort 4 was composed of patients in Cohort 1 with a new presenting case of COVID-19 diagnosis with a pre-recorded history of comorbidities such as those listed in Cohort 2. Cohort 4 was found using an additive search combination with both Cohort 1 and Cohort 2 admission codes from March 2020 - June 2021.

Race was categorized into Non-Hispanic Black or African American (Blacks, NHB), Non-Hispanic White (Whites, NHW), Native Hawaiian or other Pacific Islander (NH/PI), Asian, American Indian or Alaskan Native (AI/AN), and others and was included in the information retrieved from each Cohort.

## 3. Results

**Figure 1:**
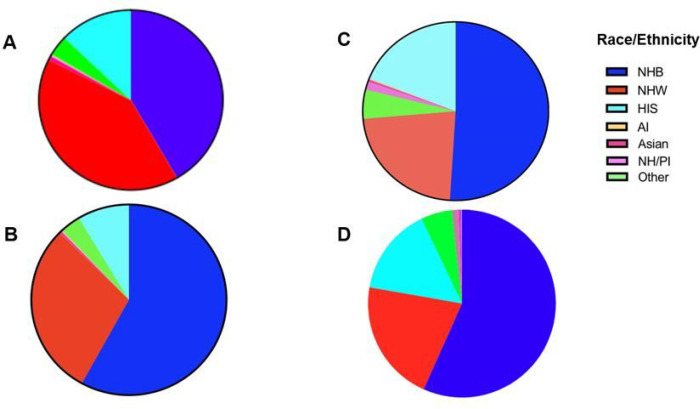
The racial and ethnic demographics of patients from Cohorts: (A) Panel IA represents the race and ethnicity of the patients admitted into Meharry Medical hospital for any disease without regard to admission type. (B) Panel IB represents the race and ethnicity of the patients admitted into Meharry Medical hospital for any disease with comorbidities without regard to admission type. (C) Panel IC represents the race of the patients admitted into Meharry Medical hospital for COVID-19 from March 2020 - June 2021., (D) Panel ID shows the racial and ethnic distribution of the patients admitted into Meharry Medical hospital for COVID-19 from March 2020 - June 2021 who presented with at least one of the studied comorbidities (HTN, DM or OBS).

For the total 237,404 patients in the Meharry system (Cohort 3), 76,596 patients were NHB, 74,998 patients were NHW, and 22,804 patients were Hispanic (HIS). The current admission rate of NHB persons based on the total admission pool was calculated to be 32.3% regardless of admission type and that of NHW persons was 31.6%. Hispanics or Latinos accounted for 10.6% of the general patient population. (Table 1, Figure 1A).

**Figure 2:**
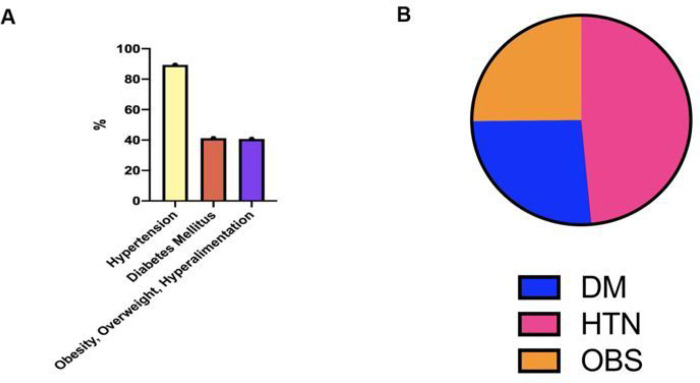
The prevalence of Hypertension, Obesity, and Diabetes of patients from Cohort 2: (A) Panel 2A denotes the percentage of patients with recorded ongoing or currently presenting HTN, DM, or OBS at Nashville General Hospital as of June 2021. (B) Panel IIB represents the proportion of patients presenting with COVID-19 who had HTN, DM, or OBS.

The majority of patients with comorbidities who visited the hospital had hypertension (HTN) (8858, 89.4%), 41.2% (4082) had Diabetes Mellitus (DM) and 40.7% (4032) had Obesity (OBS) (Figure 2A).

24,326 of the 28, 532 patients with comorbidities in Cohort 2 had demographic information. 14,111 (58.01%) were NHB. 7145 (29.37%) were NHW persons and 2091 (8.6%) were Hispanic (Figure 1B, Table 2).

Of the 959 patients with confirmed COVID-19 infection, 853 had racial demographic information. 435 (45.4%) of 959 identified as NHB persons, 193 (20.1%) identified as NHW persons, and 162 (16.9%) identified as Hispanic or Latino (Table 3 Figure 1C).

**Figure 3:**
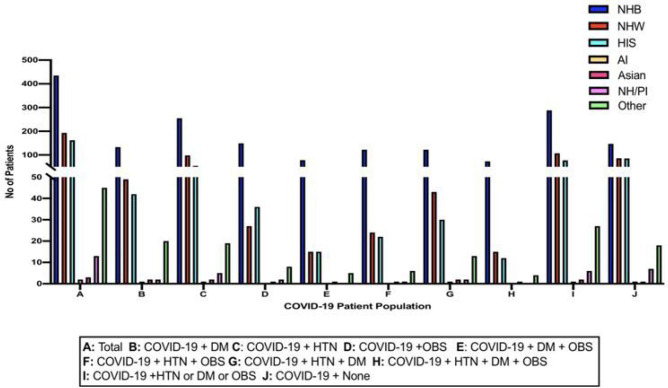
The racial and ethnic demographics from Cohort 4: This figure shows a comparison of the race and ethnicity of the patients admitted into Nashville General Hospital at Meharry for COVID-19 and HTN, OBS, and/or DM from March 2020 - June 2021.

Here, using the data set of patients from Cohort 1 (N=959) with COVID-19, a subset (Cohort 4) showed that of the COVID-19 positive population, NHB persons, 288(49.5%) had one or more comorbidities, whereas NHW persons, 107 (18%) had comorbidities (Table 3, Figure 1D). 77 (13%) of Hispanics with COVID-19 presented with comorbidities. More people in the NHB person group had both COVID-19 and comorbidities than both Hispanic and NHW populations. Within each subgroup of COVID-19 patients with comorbidities, NHB persons steadily showed higher rates of admission especially when diagnosis included OBS (Table 4, Figure 3).

The highest prevalence of singular comorbidity with COVID-19 was HTN (505, 52.7%), followed by DM (274, 28.6%) and OBS (262, 27.3%) (Supp 7, Figure VA). Many patients presented with concurrent comorbidities. HTN and DM were the most concurrent conditions (239, 24.9%), followed closely by HTN and OBS (212, 22.1%)) (Table 5, Figure 2B).

## 4. Discussion

COVID-19 has impacted the lives of many people around the world. In the United States (USA) alone, there have been over 100 million cases and 1 million deaths recorded from the virus [6]. The Centers for Disease Control (CDC) reported on February 28, 2021, an increased risk for COVID-19 infection, hospitalization, and death among varying racial groups and ethnicities in the USA. Specifically, NHB, HIS, and AI persons showed higher incidences of cases, hospitalizations, and deaths [7]. The increase is at an alarming rate, with certain populations showing almost 3-fold increases in the death rate caused by COVID-19 [7]. Two studies on COVID-19 patients from China documented clinical features and identified potential risk factors through presenting characteristics. Of the known comorbidities, hypertension (HTN) in both studies was the most common comorbidity with DM and ischemic heart disease following closely [8]. 54 of 191 patients comprising the second study expired, and it was found that those in-hospital deaths were more common in patients with cardiovascular disease and diabetes (DM) [8–10] further uncovering the role that individual comorbid conditions contribute to COVID-19 disease. In a pooled meta-analysis, HTN was found to be associated with a 2.5-fold increased risk of severe COVID-19 infection [10]. Additionally, the prevalence of HTN was also higher among those patients who died in the ICU [10]. A study conducted by Wang, on patients requiring ICU care due to COVID-19, found that ICU patients were more likely than non-ICU patients to have underlying comorbidities such as HTN, DM, and cardiovascular disease and patients with DM and Coronary Heart disease had greater odds of in-hospital death [11]. To further strengthen the associations between COVID-19 and comorbidities, Li et al found that patients with HTN were older and had more COVID-19 manifestations, such as increased number rates of respiratory distress syndrome and in-hospital mortality [8, 12]. Likewise, a case series consisting of 168 patients who died from COVID-19 found that 125 (74%) of these patients had more than one comorbidity with the most common being HTN, DM, and ischemic heart disease [8–9, 13].

Of 1,389,016 patients with DM in the USA, 97.5% of patients had at least one comorbid condition in addition to Type 2 DM and 88.5% had at least two, of which HTN was the most common (82.1%) The highest co-prevalence was HTN and hyperlipidemia (67.5%), followed closely by overweight/obesity and HTN (66.0%) [14]. Among minority groups, control rates of comorbid conditions tend to be lower. For example, HTN control rates among NHW adults (55.7%) were significantly higher than in NHB (48.5%), and Hispanic (47.4%) adults [15].

Seeing that the most common prevailing comorbidities in COVID-19 symptomatic patients are the most common comorbid conditions in the African American community, it is apparent that this sub-population is more severely affected by the COVID-19 pandemic. Hence, a knowledge of these predispositions becomes increasingly important to guide surveillance and provide patient care in the future.

These studies emphasize that not only are comorbidities a national problem but also that ethnic minority subgroups should be encouraged and provided with resources to properly care for their health as they are at an increased risk for severe infection and death owing to the greater burden of the comorbidities in their population.

Based on our findings, NHB persons showed a larger rate of contracting COVID-19 with or without the prevalence of comorbid illnesses such as HTN, DM, and OBS than NHW persons despite comparable patient visits to the hospital. In the same vein, within the patient population recorded to have comorbidities such as HTN, DM, and OBS at Nashville General Hospital, NHB persons also showed a larger rate of prevalence compared to NHW persons despite comparable patient visits to the hospital. The majority of this patient group had HTN, and a subgroup had both HTN and DM or HTN and OBS concurrently. The disparities in COVID-19 incidence become more pronounced when patients presented with OBS comorbidities either singularly or concurrently with other comorbidities. This data not only suggests that an increased incidence of COVID-19 is particularly influenced by the presence of comorbidities but also that OBS as a comorbidity is significantly associated with health disparities.

All of this begs the question: is the prevalence of comorbidities related to diet, culture, lifestyle preferences such as the presence or lack of exercise, a lack of education, poor communication in doctor visits, poor responses to follow-up, or poor adherence to doctor’s advice? The reason for this disparity needs to be fully understood to appropriate pertinent resources to such groups as comorbidities such as HTN, DM, and OBS have been shown to impact COVID-19 incidence and severity.

## 5. Conclusion

In conclusion, we show a significant association between the prevalence of comorbidities within certain ethnic groups and the incidence of COVID-19 and conclude that ethnic populations, more heavily burdened by comorbidities, are the same populations with increased mortality rates. Hence, the disproportionate presence of comorbidities in multi-racial populations, particularly African American, and increased fatality of COVID-19 among such groups emphasizes the need for addressing these issues by creating a multi-pronged approach to healthcare provision. Such an approach may include, educating healthcare providers, providing culturally sensitive care, decreasing the production of obesogens in food, marketing the importance of healthy attitudes, eliminating food deserts to increase access to better nutrition, and access to affordable healthcare in order to maintain good health because comorbidities are important factors in COVID-19 severity. These findings also suggest the need for more continued in-depth study of comorbidities and educating high risk populations on ways to maintain healthy conditions to further bridge the gap in health disparities.

## Figures and Tables

**Table 1.** Racial Demographics from Cohort 3 admitted into the Meharry Medical system for any cause Without Preference to Admission Type

Race/Ethnicity	No. of Patients	%
**NHB**	76596	32.3
**NHW**	74998	31.6
**HIS**	23647	10.6
**Other**	6753	2.8
**NH/PI**	332	0.1
**AI/AN**	344	0.1
**Asian**	1317	0.6
**Total**	237404	

**Table 2.** Racial Demographics from Cohort 2 admitted into the Meharry Medical system presenting with Disease of Circulatory System, Obesity, and Diabetes.

Race / Ethnicity	No. of Patients	%
**NHB**	14,111	58.01
**NHW**	7145	29.37
**HIS**	2091	8.60
**Other**	826	3.40
**NH/PI**	26	0.11
**Asian**	102	0.42
**AI**	25	0.10

**Table 3.** Racial Demographics admitted into the Meharry Medical system for COVID-19 and with comorbidities from March 2020 – June 2021

Race / Ethnicity		COVID-19	With comorbidity (W.C.)	
**NHB**	435	45.4%	288	56.69%
**NHW**	193	20.1%	107	21.06%
**HIS**	162	16.9%	77	15.16%
**Other**	45	4.6%	27	5.31%
**NH/PI**	13	1.4%	6	1.18%
**Asian**	3	0.3%	2	0.39%
**AI**	2	0.2%	1	0.20%
	959			

**Table 4.** Racial Demographics admitted into the Meharry Medical system for COVID-19 with Comorbidities from March 2020 - February 2021

		NHB		NHW		HIS		Asian		AI/AN		NH/PI		Other	
Patients															
COVID-19	CMDs	#	%	#	%	#	%	#	%	#	%	#	%	#	%
**+**	**HTN**	255	50	98	19	54	11	2	0.4	1	0.2	5	1	19	3.8
**+**	**DM**	133	49	49	37	42	15	2	0.7	1	0.4	2	0.7	20	7.3
**+**	**OBS**	149	57	27	10	36	14	1	0.4	0	0	2	0.8	8	3
**+**	**HTN + DM**	122	51	43	18	30	13	1	0.4	2	0.8	2	0.8	13	5
**+**	**HTN + OBS**	122	58	24	11	22	10	1	0.5	0	0	1	0.5	6	2.8
**+**	**DM + OBS**	78	59	15	11	12	9	1	0.8	0	0	0	0	5	3.8
**+**	**DM + HTN + OBS**	73	58	15	12	12	10	1	0.8	0	0	0	0	4	3.2
**+**	**DM or HTN or OBS**	288	49	107	18	77	13	2	0.3	1	0.2	6	1	27	4.6
**+**	**None**	147	39	86	23	85	23	1	27	1	0.3	7	1.9	18	5

**Table 5.** Comorbidities Distribution in COVID-19 Patients in the Meharry Medical system

Comorbidities	No of Patients	Patients
**HTN**	505	52.7
**DM**	274	28.6
**OBS**	262	27.3
**HTN + DM**	239	24.9
**HTN + OBS**	212	22.1
**DM + OBS**	133	13.9
**DM + HTN + OBS**	125	13.0
**DM or HTN or OBS**	582	60.7
**None**	377	39.3

## Data Availability

Meharry Medical College Clearsense Discover database
